# Interleukin-13 Genetic Variants, Household Carpet Use and Childhood Asthma

**DOI:** 10.1371/journal.pone.0051970

**Published:** 2013-01-30

**Authors:** Ching-Hui Tsai, Kuan-Yen Tung, Ming-Wei Su, Bor-Luen Chiang, Fook Tim Chew, Nai-Wei Kuo, Yungling Leo Lee

**Affiliations:** 1 Institute of Epidemiology and Preventive Medicine, College of Public Health, National Taiwan University, Taipei, Taiwan; 2 Research Center for Genes, Environment and Human Health, College of Public Health, National Taiwan University, Taipei, Taiwan; 3 Department of Pediatrics, National Taiwan University Hospital, Taipei, Taiwan; 4 Department of Biological Sciences, National University of Singapore, Singapore; John Hopkins Bloomberg School of Public Health, United States of America

## Abstract

*Interleukin (IL)-13* genetic polymorphisms have shown adverse effects on respiratory health. However, few studies have explored the interactive effects between *IL-13* haplotypes and environmental exposures on childhood asthma. The aims of our study are to evaluate the effects of *IL-13* genetic variants on asthma phenotypes, and explore the potential interaction between *IL-13* and household environmental exposures among Taiwanese children. We investigated 3,577 children in the Taiwan Children Health Study from 14 Taiwanese communities. Data regarding children's exposure and disease status were obtained from parents using a structured questionnaire. Four SNPs were tagged accounting for 100% of the variations in *IL-13*. Multiple logistic regression models with false-discovery rate (FDR) adjustments were fitted to estimate the effects of *IL-13* variants on asthma phenotypes. SNP rs1800925, SNP rs20541 and SNP rs848 were significantly associated with increased risks on childhood wheeze with FDR of 0.03, 0.04 and 0.04, respectively. Children carrying two copies of h1011 haplotype showed increased susceptibility to wheeze. Compared to those without carpet use and h1011 haplotype, children carrying h1011 haplotype and using carpet at home had significantly synergistic risks of wheeze (OR, 2.5; 95% CI, 1.4–4.4; p for interaction, 0.01) and late-onset asthma (OR, 4.7; 95% CI, 2.0–10.9; p for interaction, 0.02). In conclusions, *IL-13* genetic variants showed significant adverse effects on asthma phenotypes among children. The results also suggested that asthma pathogenesis might be mediated by household carpet use.

## Introduction

Asthma not only results in morbidity or school absence in school children [1], [2] but also leads to raising medical costs and social burden [3]. The prevalence of childhood asthma/wheeze has been reported as increasing globally [4], [5]. Diverse genetic and environmental components have been noted for this complex disease. Recent studies have suggested that many genetic variants were associated with childhood asthma-related diseases that occur when environmental factors trigger immune responses [6], [7].

Interleukin (IL)-13 is an important T-helper type 2 (Th_2_) cytokine involved in the inflammation of asthmatic airways [8]. In animal models, pulmonary expression of IL-13 was reported to include eosinophilic tissue inflammation, subepithelial fibrosis, mucus hypersecretion and airway hyperresponsiveness (AHR) to methacholine [9], [10]. Many epidemiological studies also revealed that the variants in the *IL-13* gene were associated with total IgE level, increased eosinophil count, atopy and asthma among children [11], [12], [13], [14].

The most important risk factor in the development of allergic diseases such as asthma is induction of IgE against indoor allergens, and imbalance between T-helper type 1 (Th_1_) and Th_2_ cytokine responses for skewing to Th_2_ response [15]. Household carpet use is known to be a reservoir of major indoor allergens [16], which have been suggested to increase airway inflammation and asthma in children [17], [18]. Previous studies have shown that household environmental tobacco smoke (ETS) and *IL-13* genetic variants may have interactive effects on asthma phenotypes [19], [20]. However, there is no related literature concerning the association between childhood exposure to household carpet use and *IL-13* genetic polymorphisms that might be involved in asthma susceptibility. Haplotype analyses of *IL-13* were also unclear among the Chinese population.

Taiwan Children Health Study (TCHS) is a population-based study representing a wide range of environmental factors and genetic susceptibility. TCHS offers an opportunity to investigate the gene-environment interactive effects on respiratory health. In present study, we explored the associations between *IL-13* genotypes/haplotypes, household carpet use and asthma phenotypes among children.

## Materials and Methods

### Study population

We conducted a population-based survey for children's health in 2007 and the study protocol has been described in detail previously [21], [22]. Briefly, TCHS recruited 5,082 middle-school children from 14 diverse communities in Taiwan. The parents or guardians of each participating student provided written informed consent at study entry. In this analysis, we randomly selected 3,577 seventh-grade children who provided their buccal cells as the DNA resource for genotyping, and there were no differences in the main characteristics between genotyped and non-genotyped subjects. The study protocol was approved by the Institutional Review Board.

### Questionnaire of asthma phenotypes

The standard questionnaire for childhood exposures and health status was taken home by students and answered by parents or guardians. Children were considered to have asthma if there was a positive answer to the question “Has a doctor ever diagnosed this child as having asthma?” Wheeze was defined as any occurrence of the child's chest sounding wheezy or whistling. Early-onset asthma was defined as age of onset for asthma before 5 years of age. Late-onset asthma was onset after 5 years of age.

### Exposure assessment and covariates

Household carpet use exposure was determined from the question “Have you ever used carpets in the living room, children's bedroom or other bedrooms in your house?” Basic demographic data and possible confounding variables were also collected, including sex, age, community, personal/family history of asthma or atopic diseases, *in utero* exposures to maternal smoking, ETS, dampness, incense burning, and pet ownership at home.

### SNPs selection

**Figure 1:**
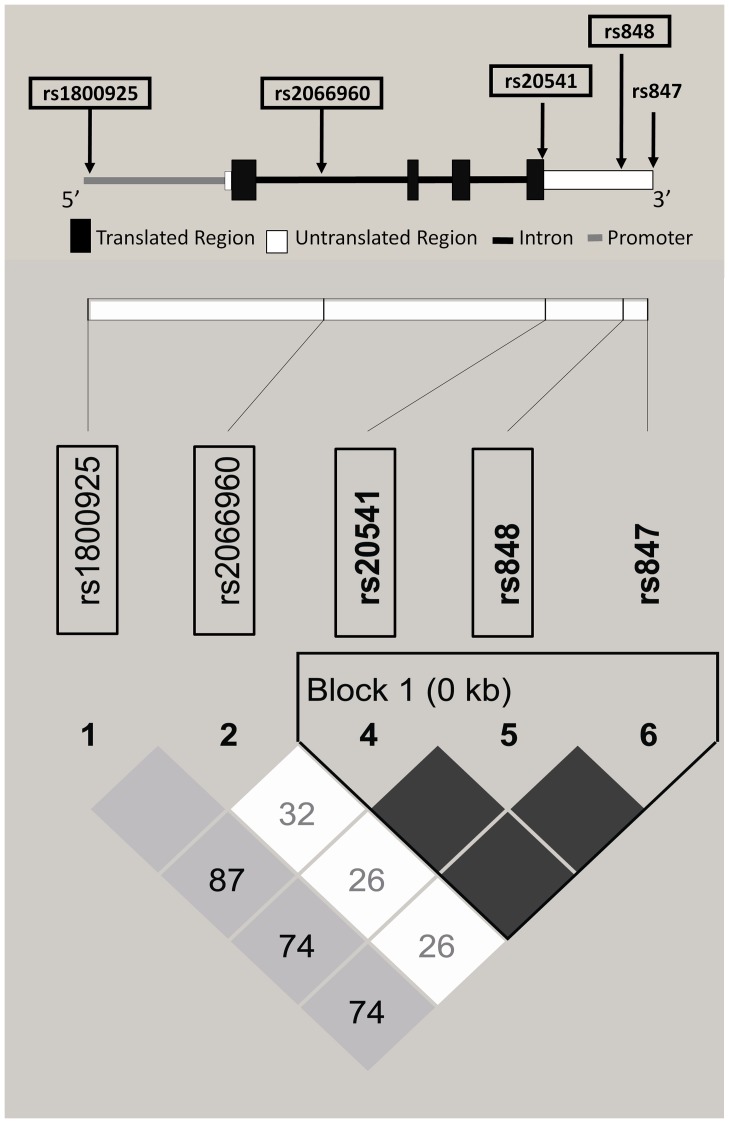
The location and LD of *IL-13* SNPs were plotted. D' is pairwise linkage disequilibrium using the Haploview program. The block structure of *IL-13* is defined by the confidence intervals of D'. D' values are displayed in the lozenge-shaped cells, and empty cells indicate D' = 1. Four SNPs (squared) are selected and they accounted for 100% of the variations in *IL-13*.

A list of *IL-13* SNPs was provided by the Han Chinese in Beijing genome panel (CHB_GENO_PANEL) of Environmental Genome Project of National Institute of Environmental Health Sciences (NIEHS) (http://egp.gs.washington.edu/). We studied the genomic region of the *IL-13* and the region 2000 bp upstream of the gene. All SNPs were used as input files for the Haploview v4.1 (http://www.broadinstitute.org/mpg/haploview) to select tag SNPs and to investigate the linkage disequilibrium (LD) patterns for *IL-13*. One of the six SNPs was excluded due to minor allele frequency (MAF) less than 0.05 [23], [24]. As shown in Fig. 1, one haplotype block in strong LD was defined by the confidence intervals of D' (where the upper CI limit was 0.98 and the lower CI limit was 0.70) [25]. Tagging SNPs were selected because of pairwise tagging tests at the prescribed squared correlation (r^2^) value ≧0.95 [24]. Pairwise tagging tests simply apply tag SNP represented to all the other SNPs, due to their highly correspondence [26]. The four tag SNPs of *IL-13* were SNP rs1800925, SNP rs2066960, SNP rs20541 and SNP rs848, and they captured 100% of allele's variations in *IL-13*.

### DNA collection and genotyping

Genomic DNA was isolated from cotton swabs containing oral mucosa using phenol/chloroform extraction method [27]. The TaqMan assays were performed using a TaqMan PCR Core Reagent kit (Applied Biosystems, Foster City, CA) according to manufacturer's instructions. PCR amplification using 1.0∼5.0 ng of genomic DNA was performed with an initial step of 95°C for 5 min followed by 40 cycles of 95°C for 30 s and 60°C for 30 s. The fluorescence profile of each well was measured in an ABI 7900HT Sequence Detection System (Applied Biosystems, Foster City, CA) and the results analyzed with Sequence Detection Software (Applied Biosystems, Foster City, CA). Experimental samples were compared with 36 controls to identify the three genotypes at each locus. Any samples that were outside the parameters defined by the controls were identified as non-informative and were retested. We also repeated 15% of randomly selected DNA samples to verify and confirm the results of genotyping for four *IL-13* SNPs. The details of primer and probe sequences were presented in table S1. The genotype completion rates were between 97% and 99% for all loci.

### Statistical analysis

Unconditional multiple logistic regression models were fitted to estimate the individual effects of *IL-13* on asthma phenotypes. Models are adjusted for age, sex, parental history of asthma, parental history of atopy, *in utero* exposures to maternal smoking, ETS, dampness, incense burning, pet ownership at home and community. When considering the effects about *IL-13* additive genetic model was utilized. Selection of confounders that were included in the model was based on *a priori* consideration and the standard statistical procedure of 10% change in point estimates [28]. Subjects with missing covariate information were included in the model using missing indicators [29]. Estimates of measurements of LD and r^2^ in TCHS participants were obtained from Haploview.

Haplotype frequencies were estimated from the *IL-13* genotype data using the EM algorithm by TagSNPs [30]. The likelihood ratio test (LRT) was used to detect the global association of four variants of *IL-13* haplotypes with asthma phenotypes. We collapsed rare haplotypes (frequency <0.05) into a category in the log additive haplotype analyses. The numbers of copies of each haplotype a person carries and appropriate confidence intervals were estimated using a single imputation procedure [30]. Logistic regression models were based on the co-dominant (two copies of the haplotype vs. no copy and one copy of the haplotype vs. no copy) and dominant (at least one copy of the haplotype vs. no copy) inheritance model. We estimated the false-discovery rate (FDR) for multiple comparisons for main effect associations [31], and used FDR <0.05 as a criterion.

The interaction between genotype or haplotype and household carpet use was assessed by adding an interactive term in the logistic regression model and a likelihood ratio test was used to test its significance. All analyses were conducted using SAS software version 9.1 (SAS Institute, Cary, NC, USA).

## Results

A total of 3,577 children with genotyping data were enrolled in current study. The mean age of participants was 12.3±0.5 years and all of them were of ethnic Han Chinese origin (Table 1). Among these children, 8.7% live in households with carpets, and 43.4% have ETS exposure at home. The prevalence of asthma was 8.0% and wheeze occurred in 12.1% of participants.

**Table 1:** Selected characteristics for participants in Taiwan Children Health Study.

	With genotyping		All eligible participants	
	(N = 3577)		(N = 5082)	
	N	%	N	%
***Demographic information***				
Sex				
Boy	1755	49.1	2464	48.5
Girl	1822	50.9	2618	51.5
Age, yr (Mean ± SD)	12.26±0.50		12.42±0.65	
Parental asthma†				
No	3335	96.9	4739	97.1
Yes	108	3.1	140	2.9
Parental atopy†				
No	2536	73.7	3617	74.1
Yes	907	26.3	1262	25.9
***Home exposures*** †				
Carpet use	308	8.7	442	8.7
*In utero* exposures to maternal smoking	142	4.0	198	3.9
ETS at home	1544	43.4	2265	44.9
				
***Respiratory Outcomes*** †				
Asthma	284	8.0	375	7.4
Wheeze	431	12.1	586	11.6
Early-onset asthma‡	184	5.3	239	4.9
Late-onset asthma§	92	2.7	123	2.6

† Number of subjects do not add up to total N because of missing data.

‡ Early-onset: asthma diagnosed ≦5 yr of age.

§ Late-onset: asthma diagnosed >5 yr of age.

The genotype frequencies of *IL-13* were showed in Table 2 and distributions of the four selected SNPs were in Hardy-Weinberg equilibrium (p value cutoff >10^−4^) [23]. The pairwise measures of LD for *IL-13* were presented in Table S2 and we could find SNP rs20541 and SNP rs848 in a strong LD.

**Table 2:** Genotype frequencies of *IL-13* in this study participants.

	N	%
IL-13 genotypes		
SNP rs1800925		
CC	2750	73.6
CT	877	23.5
TT	109	2.9
Minor allele frequency	0.15	
HWE	8×10^−4^	
SNP rs2066960		
CC	1428	37.6
CA	1725	45.4
AA	645	17.0
Minor allele frequency	0.40	
HWE	0.01	
SNP rs20541		
CC	1774	46.5
CT	1653	43.3
TT	391	10.2
Minor allele frequency	0.33	
HWE	0.86	
SNP rs848		
GG	1718	45.9
GT	1599	42.7
TT	427	11.4
Minor allele frequency	0.33	
HWE	0.08	

HWE: Hardy-Weinberg equilibrium.

After adjustment for age, sex, parental history of asthma, parental history of atopy, *in utero* exposures to maternal smoking, ETS, dampness, incense burning, pet ownership at home and community, *IL-13* SNPs showed statistical significance for the occurrence of wheeze (FDR<0.05) (Table 3 and Table S3). Children carrying T allele of SNP rs1800925 were associated with increased risks on wheeze (OR, 1.3; 95% CI, 1.1–1.5; FDR, 0.03). The variant allele of SNP rs2066960 was protective for wheeze (OR, 0.9; 95% CI, 0.7–1.0; FDR, 0.04). SNP rs20541 and SNP rs848 revealed a similar associated pattern with asthma phenotypes, the increased risks of wheeze were related to children with T allele in additive models.

**Table 3:** Association of IL-13 with asthma phenotypes, by additive genetic model.

SNP	Asthma				Wheeze				Early-onset asthma†				Late-onset asthma‡			
	OR	95% CI	P value	FDR	OR	95% CI	P value	FDR	OR	95% CI	P value	FDR	OR	95% CI	P value	FDR
rs1800925	1.2	(0.9,1.5)	0.21	0.41	1.3	(1.1,1.5)	0.01	0.03	1.2	(0.9,1.5)	0.23	0.60	1.2	(0.8,1.7)	0.41	0.76
rs2066960	0.9	(0.7,1.1)	0.12	0.41	0.9	(0.7,1.0)	0.02	0.04	1.0	(0.8,1.2)	0.60	0.60	0.7	(0.5,1.0)	0.03	0.13
rs20541	1.1	(0.9,1.3)	0.45	0.49	1.2	(1.0,1.4)	0.04	0.04	1.1	(0.9,1.4)	0.44	0.60	1.1	(0.8,1.5)	0.66	0.76
rs848	1.1	(0.9,1.3)	0.49	0.49	1.2	(1.0,1.4)	0.04	0.04	1.1	(0.9,1.3)	0.54	0.60	1.0	(0.8,1.4)	0.76	0.76

Models are adjusted for age, sex, parental history of asthma, parental history of atopy, *in utero* exposures to maternal smoking, ETS, dampness, incense burning, pet ownership at home and community.

† Early-onset: asthma diagnosed ≦5 yr of age.

‡ Late-onset: asthma diagnosed >5 yr of age.

Haplotype frequencies of the four SNPs were presented in Table 4, and the h0100 was the most common haplotype. The globe haplotype association using likelihood ratio test with 4 degrees of freedom was only significant for wheeze (Table 5). Compared with h0100 haplotype, children with h1011 haplotype had a significantly increased risk of wheeze (OR, 1.5; 95% CI, 1.2–1.8). The h0000 haplotype was correlated with late-onset asthma (OR, 1.5; 95% CI, 1.0–2.2).

**Table 4:** Haplotype frequencies of *IL-13* in this study participants.

Haplotype^a^	frequency
h0100	0.3314
h0000	0.3088
H0011	0.1356
H1011	0.1133
H0111^b^	0.0470
H0001^b^	0.0173
H1000^b^	0.0135
H1100^b^	0.0083
H0010^b^	0.0079
H1111^b^	0.0057
H1010^b^	0.0039
H0101^b^	0.0033
H0110^b^	0.0014
H1001^b^	0.0012
H1101^b^	0.0010
H1110^b^	0.0004

a 0: common allele and 1: minor allele, by the order of SNP1 (rs1800925): C/T; SNP2 (rs2066960): C/A; SNP3 (rs20541): C/T; SNP4 (rs848): G/T.

b Haplotypes were collapsed into a category in the haplotype analyses.

**Table 5:** Association of *IL-13* haplotypes with asthma phenotypes among children.

	Asthma		Wheeze		Early-onset asthma†		Late-onset asthma‡	
	OR	95%CI	OR	95%CI	OR	95%CI	OR	95%CI
h0100^a^	1		1		1		1	
h0000^a^	1.1	(0.9,1.4)	1.1	(0.9,1.4)	1.0	(0.7,1.3)	**1.5**	**(1.0,2.2)**
h0011^a^	1.2	(0.9,1.6)	1.2	(0.9,1.5)	1.2	(0.8,1.6)	1.3	(0.8,2.1)
h1011^a^	1.2	(0.9,1.7)	**1.5**	**(1.2,1.8)**	1.2	(0.8,1.7)	1.5	(0.9,2.4)
Others	0.8	(0.6,1.2)	1.0	(0.8,1.4)	0.7	(0.5,1.2)	1.0	(0.5,1.9)
globe p value	0.22		**0.03**		0.34		0.21	

Models are adjusted for age, sex, parental history of asthma, parental history of atopy, *in utero* exposures to maternal smoking, ETS, dampness, incense burning, pet ownership at home and community.

† Early-onset: asthma diagnosed ≦5 yr of age.

‡ Late-onset: asthma diagnosed >5 yr of age.

a 0: common allele and 1: minor allele, by the order of SNP1 (rs1800925): C/T; SNP2 (rs2066960): C/A; SNP3 (rs20541): C/T; SNP4 (rs848): G/T.

The *IL-13* haplotype copy numbers analyses were presented in Table 6. Compared with children without h1011 haplotype, those with two copies of h1011 haplotype showed a significantly 2.4-fold increased risk of wheeze (95% CI, 1.3–4.5; FDR, 0.04). It also showed a dose-response relationship on the increased risks, with OR 1.3 (95% CI, 1.0–1.6; FDR, 0.23) for those with one copy of h1011 haplotype, and OR 2.4 (95% CI, 1.3–4.5; FDR, 0.04) for those with two copies.

**Table 6:** Association of *IL-13* haplotype copy numbers with wheeze among children.

	OR	95%CI	P value	FDR		OR	95%CI	P value	FDR
h0100^a^					h0011^a^				
0 copies	1				0 copies	1			
1 copy	0.9	(0.7,1.1)	0.19	0.39	1 copy	1.1	(0.9,1.5)	0.41	0.65
2 copies	0.7	(0.5,1.0)	0.04	0.19	2 copies	1.1	(0.6,2.2)	0.75	0.89
≧1 copy	0.8	(0.7,1.0)	0.07	0.14	≧1 copy	1.1	(0.9,1.5)	0.39	0.52
h0000^a^					h1011^a^				
0 copies	1				0 copies	1			
1 copy	1.0	(0.8,1.3)	0.86	0.89	1 copy	1.3	(1.0,1.6)	0.08	0.23
2 copies	1.0	(0.7,1.4)	0.89	0.90	2 copies	2.4	(1.3,4.5)	0.01	0.04
≧1 copy	1.0	(0.8,1.3)	0.92	0.92	≧1 copy	1.4	(1.1,1.7)	0.02	0.07

Models are adjusted for age, sex, parental history of asthma, parental history of atopy, *in utero* exposures to maternal smoking, ETS, dampness, incense burning, pet ownership at home and community.

a 0: common allele and 1: minor allele, by the order of SNP1 (rs1800925): C/T; SNP2 (rs2066960): C/A; SNP3 (rs20541): C/T; SNP4 (rs848): G/T.

Some household environmental factors were investigated in the TCHS (Table S4). We found no substantial differences on the effect of the *IL-13* genetic variants among children in relation to exposure to the other environmental factors, such as ETS, dampness, incense burning, and pet ownership at home. The association of household carpet use and ETS with asthma phenotypes was shown in Table S5. Children in households with carpet use had an increased risk on late-onset asthma (OR, 1.8; 95% CI, 1.0–3.3; FDR, 0.07). To further investigate the interactions of household factors and *IL-13* variants on children's respiratory health, we examined the relationships of household carpet use with asthma phenotypes (Table 7). Joint exposure appeared to increase the individual effects of SNP rs1800925 and household carpet use on wheeze (OR, 2.0; 95% CI, 1.2–3.6; p for interaction, 0.03) and late-onset asthma (OR, 3.9; 95% CI, 1.7–9.1; p for interaction, 0.04). SNP rs20541 and SNP rs848 and household carpet use showed synergistic effects on late-onset asthma, with the OR 2.5 (95% CI, 1.2–5.2; p for interaction, 0.03) for those with T allele in SNP rs20541 and household carpet use, and with the OR 2.6 (95% CI, 1.2–5.3; p for interaction, 0.02) for those with T allele in SNP rs848 and household carpet use.

**Table 7:** Joint effects of carpet use and *IL-13* genotypes on asthma phenotypes among children.

		Asthma		Wheeze		Early-onset asthma†		Late-onset asthma‡	
	Carpet use	OR	95%CI	OR	95%CI	OR	95%CI	OR	95%CI
SNP rs1800925									
CC	No	1		1		1		1	
CC	Yes	0.7	(0.4,1.2)	0.7	(0.5,1.2)	0.5	(0.2,1.2)	1.1	(0.5,2.6)
CT or TT	No	1.1	(0.8,1.5)	1.2	(0.9,1.5)	1.2	(0.8,1.7)	1.0	(0.6,1.7)
CT or TT	Yes	1.4	(0.7,3.0)	2.0	(1.2,3.6)	0.5	(0.1,1.9)	3.9	(1.7,9.1)
P for interaction		0.17		0.03		0.75		0.04	
SNP rs2066960									
CC	No	1		1		1		1	
CC	Yes	0.8	(0.3,1.9)	1.3	(0.7,2.3)	0.6	(0.2,2.1)	1.1	(0.3,3.7)
CA or AA	No	1.0	(0.7,1.2)	0.9	(0.7,1.1)	1.1	(0.8,1.6)	0.7	(0.4,1.0)
CA or AA	Yes	0.8	(0.5,1.5)	0.8	(0.5,1.3)	0.4	(0.2,1.1)	1.5	(0.7,3.1)
P for interaction		0.84		0.30		0.52		0.28	
SNP rs20541									
CC	No	1		1		1		1	
CC	Yes	0.6	(0.3,1.3)	0.7	(0.4,1.3)	0.6	(0.3,1.6)	0.7	(0.2,2.4)
CT or TT	No	1.0	(0.8,1.3)	1.1	(0.9,1.4)	1.2	(0.9,1.6)	0.8	(0.5,1.3)
CT or TT	Yes	1.1	(0.6,2.0)	1.4	(0.9,2.3)	0.4	(0.1,1.2)	2.5	(1.2,5.2)
P for interaction		0.30		0.15		0.36		0.03	
SNP rs848									
GG	No	1		1		1		1	
GG	Yes	0.6	(0.3,1.3)	0.7	(0.4,1.3)	0.6	(0.2,1.6)	0.7	(0.2,2.2)
GT or TT	No	1.0	(0.8,1.3)	1.2	(0.9,1.4)	1.2	(0.9,1.6)	0.8	(0.5,1.2)
GT or TT	Yes	1.1	(0.6,2.0)	1.5	(0.9,2.4)	0.4	(0.1,1.2)	2.6	(1.2,5.3)
P for interaction		0.26		0.13		0.39		0.02	

Models are adjusted for age, sex, parental history of asthma, parental history of atopy, *in utero* exposures to maternal smoking, ETS, dampness, incense burning, pet ownership at home and community.

† Early-onset: asthma diagnosed ≦5 yr of age.

‡ Late-onset: asthma diagnosed >5 yr of age.

Because the effect of h1011 was greater than other haplotypes (Table 5), we investigated the association between h1011 haplotype, household carpet use and asthma phenotypes. Compared to those without household carpet use and h1011 haplotype, children carrying h1011 haplotype and living in homes with carpets had increased risks of wheeze (OR, 2.5; 95% CI, 1.4–4.4; p for interaction, 0.01) and late-onset asthma (OR, 4.7; 95% CI, 2.0–10.9; p for interaction, 0.02) (Table 8). However, we could not find the similar pattern for ETS at home on the effects of h1011 haplotype to asthma phenotypes in childhood (Table S6).

**Table 8:** Joint effects of carpet use and *IL-13* haplotype h1011 on asthma phenotypes among children.

		h1011^a^				
		No		Yes		
	Carpet use	OR	95%CI	OR	95%CI	P for interaction
Asthma	No	1		1.1	(0.8,1.4)	0.06
	Yes	0.6	(0.3,1.1)	1.7	(0.8,3.6)	
Wheeze	No	1		1.2	(0.9,1.5)	0.01
	Yes	0.7	(0.4,1.1)	2.5	(1.4,4.4)	
Early-onset asthma†	No	1		1.1	(0.8,1.6)	0.98
	Yes	0.5	(0.2,1.1)	0.5	(0.1,2.2)	
Late-onset asthma‡	No	1		1.0	(0.6,1.7)	0.02
	Yes	1.0	(0.4,2.4)	4.7	(2.0,10.9)	

Models are adjusted for age, sex, parental history of asthma, parental history of atopy, *in utero* exposures to maternal smoking, ETS, dampness, incense burning, pet ownership at home and community.

† Early-onset: asthma diagnosed ≦5 yr of age.

‡ Late-onset: asthma diagnosed >5 yr of age.

a 0: common allele and 1: minor allele, by the order of SNP1 (rs1800925): C/T; SNP2 (rs2066960): C/A; SNP3 (rs20541): C/T; SNP4 (rs848): G/T.

## Discussion

To the best of our knowledge, this is the first study concerning the potential interactive associations between *IL-13*, household carpet use and childhood asthma. In our data, *IL-13* genetic variants showed significant adverse effects on asthma phenotypes. We found that children carrying h1011 haplotype have increased risks of the occurrence of wheeze. Household carpet use appears to modify the effects of *IL-13* gene on wheeze and late-onset asthma.

The *IL-13* gene is located on chromosome 5q, which has been suggested to be associated with the risk of elevated serum IgE levels, eosinophilia, airway hyper-sensitiveness and the occurrence of childhood asthma [13], [14]. In our study, children with variant alleles of SNP rs1800925, SNP rs20541 and SNP rs848 have significantly higher risks for wheeze (Table 3). Previous studies have shown that promoter variant in *IL-13* (SNP rs1800925) could enhance *IL-13* transcription [32] and was associated with atopy, asthma and increased bronchial hyper-reactivity [13], [33], [34]. Another polymorphism in exon4 (SNP rs20541, Arg130Gln) also resulted in the occurrence of asthma, reduced lung function and increased serum IgE [13], [35], [36]. Functional and association studies both showed that 130Gln was related to higher IL-13 levels and a stronger Th_2_ immune response than 130Arg [37]. Few studies focused on rs848 polymorphism with asthma. Hunninghake *et al*. have indicated that SNP rs848 was in strong linkage disequilibrium with SNP rs20541 [13], which is consistent with our findings (Fig. 1 and Table S2). However, the effects of SNP rs2066960 variants in previous studies showed that minor allele (A allele) was associated with elevated serum IgE and early-transient wheeze [19], [38]. In our data, we found that SNP rs2066960 A allele was associated with decreased risks of asthma phenotypes (Table 3). The different genetic association on asthma might be attributed to genotype frequencies in different ethnic populations. Based on the haplotype analyses in the present study, we found that three variants of 4 SNPs in *IL-13* gene might significantly affect risks of asthma phenotypes in children. Compared with the common haplotype, children carrying h1011 haplotype were more susceptible to development of wheeze (OR, 1.5; 95% CI, 1.2-1.8) (Table 5). Furthermore, the more copy numbers of the h1011 haplotype children carry, the higher risk of wheeze they would possess (Table 6).

IL-13, independent from IL-4, plays a central role for the development of asthma-related symptoms in animal models and human studies [10], [39]. IL-13 was primarily produced by Th_2_ CD4^+^ T cells after allergen irritation, and it may induce the entire pathogenic pathway of asthma independently of traditional cells, such as mast cells and eosinophils [8]. Genetic variants in *IL-13* have been found to be associated with elevated serum levels of IL-13 [37]. Household carpet use is a significant reservoir of allergens, including house dust mite, dog and cat dander, and fungal concentrations [17], [40]. House dust mites, *Dermatophagoides pteronyssinus* allergen 1 (*Der p* 1) and *Dermatophagoides farinae* (*Der f* 1), might play important roles in allergic sensitization, as well as in the development of asthma and asthma deterioration in children [41], [42], [43]. In our data, the interactive effects are consistent with *IL-13* genotypes and household carpet use on asthma phenotypes (Table 7). Especially in SNP rs1800925, joint exposure appeared to increase the individual effects of SNP rs1800925 T allele and household carpet use on wheeze (OR, 2.0; 95% CI, 1.2–3.6) and late-onset asthma (OR, 3.9; 95% CI, 1.7–9.1). It was suggested that the variant of promoter region, containing a binding site of the nuclear factor of activated T cells (NFAT) transcription factor, regulates *IL-13* and *IL-4* gene expression [33]. Additionally, our results indicated that children with h1011 haplotype and exposure to carpets may have increased risks for asthma phenotypes (Table 8). We believe that *IL-13* genetic variants and exposure to household carpet use may synergistically induce high IL-13 levels to inflammation, which would result in the occurrence of asthma phenotypes in children.

Up to the present, two studies concerning *IL-13*-environmental interaction on asthma phenotypes in children have been reported. Sadeghnejad and colleagues investigated SNP rs1800925, SNP rs2066960 and SNP rs20541 and demonstrated that the effect of ETS exposure at home was stronger on wheeze with the common *IL-13* haplotype compared to those without it [19]. Sorensen *et al*. reported that children exposed to maternal smoking during pregnancy and with SNP rs20541 C allele had increased risks on wheeze [20]. However, no significant interaction between *IL-13* polymorphisms (SNP rs20541 and SNP rs1800925) and ETS exposure at home were noted. In our data, we could not find interactive effects between *IL-13* and ETS exposure at home in asthma phenotypes (Table S6). Several reasons, including differences in ethnicity or genotype frequencies, may explain this situation. For example, the genotype frequencies in SNP rs2066960 were 81.5, 17.6% and 0.9% for CC, CA and AA genotypes, respectively, in British population [19]. In SNP rs20541, the genotype frequencies were 61.8∼64.4%, 32.1∼33.1%, and 3.5∼5.1% for CC, CT and TT genotypes, respectively, in European populations [19], [20]. Our study showed distinctly different results: 37.6%, 45.4% and 17.0% for CC, CA and AA genotypes in SNP rs2066960, and 46.7%, 44.4% and 8.9% for CC, CT and TT genotypes in SNP rs20541 (Table 2). All of the results were similar to ethnic Han Chinese in the Beijing population from HapMap (data not shown). Our population did provide evidence for *IL-13* genetic variants on asthma phenotypes among Han children.

Age, sex, parental atopic history, maternal smoking during pregnancy, ETS, dampness, incense burning, and pet ownership at home were believed to contribute to asthma and wheeze in childhood [4], [21], [44], [45], [46]. We minimized interference from these confounders by recruiting lifelong non-smokers of similar age, and adjusting potential confounders by regression models. Difference in participation by children with respiratory outcomes who had different carpet exposure histories is unlikely to be significant enough to produce substantial bias, as participation rates in each classroom were high and the characteristics was similar between genotyping and all participants (Table 1). Because the differences in distribution are modest and are probably not associated with the genotypes, it is unlikely that selection of subjects biased the effect estimates in our results. As the study subjects were recruited in an unselected population, unbiased observations of the association between genetic effects and outcomes were expected.

Our definition of household carpet use might not indicate a good quantitative biomarker for measuring allergen levels in the houses. However, higher concentrations of indoor allergens have been reported to be associated with carpet use at home [16], [47]. Exposure assessment from the questionnaire was likely to introduce some misclassification bias shifting the results toward the null. Moreover, the associations between household carpet use and childhood asthma were consistent in another case-control study from our group [18]. Another possible limitation is recall bias of respiratory outcomes. Asthma phenotypes in our study were ascertained by parental-reported questionnaire, so misclassification may have arisen from imperfect parental recall of events. Differential misclassification by *IL-13* genetic variants was probably not a major source of bias that accounts for our results, because disease status was defined without the knowledge of genotype. We found that large parts of significant effects were limited on wheeze and effect estimates of wheeze were also stronger than other asthma phenotypes. We believe that wheeze is the most common respiratory symptom in children when occurrence of airway inflammation induced by environmental stimuli. In Taiwan, parents of children with wheeze symptoms might be unlikely to seek medical care, and therefore physician-diagnosed asthma would be underreported. Consistent with previous well-known knowledge, we found the significant genetic association in *IL-13* gene, and wheeze is a good predictor for development of asthma in children.

In conclusions, our results showed that genetic variants in *IL-13*, especially h1011 haplotype, showed adverse effects on respiratory health in children. Household carpet use may influence the severity of diverse allergic inflammatory reactions induced by *IL-13* genetic variants. Additional long-term research is necessary to explore the roles played by other genes in determining genetic susceptibility on adverse respiratory outcomes. Identification of gene-environmental interactions in childhood asthma may lead to new and comprehensive insights into asthma pathogenesis and treatment.

## Supporting Information

Table S1 **Primer and probe sequences for** ***IL-13*** **genetic variants.**
(DOC)

Table S2 **Pairwise measures of linkage disequilibrium for** ***IL-13*** **in this study participants.**
(DOC)

Table S3 **Association of** ***IL-13*** **genotypes with asthma phenotypes, by co-dominant and dominant genetic model.**
(DOC)

Table S4 **Environmental questions of TCHS questionnaire.**
(DOC)

Table S5 **Association of household carpet use and ETS at home with asthma phenotypes among children.**
(DOC)

Table S6 **Joint effects of ETS exposure and** ***IL-13*** **haplotype h1011 on asthma phenotypes among children.**
(DOC)
