# Supplementary material for: Interleukin-13 Genetic Variants, Household Carpet Use and Childhood Asthma
Source: PLoS One. 2013 Jan 30;8(1):e51970. doi: 10.1371/journal.pone.0051970 (PMC3559736; doi:10.1371/journal.pone.0051970)
Supplement: Table S1 — Primer and probe sequences for IL-13 genetic variants. (DOC) [file pone.0051970.s001.doc]

Table S1. Primer and probe sequences for *IL-13* genetic variants

| SNPs | Sequence |
| --- | --- |
| SNP rs1800925 |  |
| Forward primer | 5’-ACACCCAACAGGCAAATGC-3’ |
| Reverse primer | 5’-TGTCGCCTTTTCCTGCTCTT-3’ |
| Probe 1 | 5’-(VIC) ACTTCTAGGAAAATGAG -3’ |
| Probe 2 | 5’-(6FAM) ACTTCTAGGAAAACGAG -3’ |
| SNP rs2066960 |  |
| Forward primer | 5’- GCATTTGCCAACTGGATTTT-3’ |
| Reverse primer | 5’- GGCAAGGAGCGGACTCTAC-3’ |
| Probe 1 | 5’- (VIC) AAGGGCGGGCCTATTA-3’ |
| Probe 2 | 5’-(6FAM) AAGGGCGGGCCTATGA -3’ |
| SNP rs20541 |  |
| Forward primer | 5’- CCTGTCTCTGCAAATAATGATGCT-3’ |
| Reverse primer | 5’- GGTGGCCCAGTTTGTAAAGG-3’ |
| Probe 1 | 5’- (VIC) AGTTGAACTGTCCCTC-3’ |
| Probe 2 | 5’-(6FAM) AAGTTTCAGTTGAACCGT-3’ |
| SNP rs848 |  |
| Forward primer | 5’-AATTACTCGTTGGCTGAGAGCTG-3’ |
| Reverse primer | 5’-GCAACTGAGGCAGACAGCAG-3’ |
| Probe 1 | 5’-(VIC) CAGGGACTCCTGGGGTCCACTG -3’ |
| Probe 2 | 5’-(6FAM) CAGGGACTCCTGGTGTCCACTGCT -3’ |
