# Supplementary material for: Interleukin-13 Genetic Variants, Household Carpet Use and Childhood Asthma
Source: PLoS One. 2013 Jan 30;8(1):e51970. doi: 10.1371/journal.pone.0051970 (PMC3559736; doi:10.1371/journal.pone.0051970)
Supplement: Table S2 — Pairwise measures of linkage disequilibrium for IL-13 in this study participants. (DOC) [file pone.0051970.s002.doc]

| Table S2. Pairwise measures of linkage disequilibrium for *IL-13* in this study participants | | | | |
| --- | --- | --- | --- | --- |
| D' \ r^2^ | SNP1 (rs1800925) | SNP2 (rs2066960) | SNP3 (rs20541) | SNP4 (rs848) |
| SNP1 (rs1800925) | - | 0.06^a^ | 0.21^a^ | 0.19^a^ |
| SNP2 (rs2066960) | 0.72^b^ | - | 0.10^a^ | 0.10^a^ |
| SNP3 (rs20541) | 0.76^b^ | 0.57^b^ | - | 0.84^a^ |
| SNP4 (rs848) | 0.73^b^ | 0.56^b^ | 0.93^b^ | - |
| ^a^r^2^: upper triangle area. ^b^D': lower triangle area. | | | | |
