# Supplementary material for: Interleukin-13 Genetic Variants, Household Carpet Use and Childhood Asthma
Source: PLoS One. 2013 Jan 30;8(1):e51970. doi: 10.1371/journal.pone.0051970 (PMC3559736; doi:10.1371/journal.pone.0051970)
Supplement: Table S3 — Association of IL-13 genotypes with asthma phenotypes, by co-dominant and dominant genetic model. (DOC) [file pone.0051970.s003.doc]

| Table S3. Association of *IL-13* genotypes with asthma phenotypes, by co-dominant and dominant genetic model | | | | | | | | | | | | | | | | | | | |
| --- | --- | --- | --- | --- | --- | --- | --- | --- | --- | --- | --- | --- | --- | --- | --- | --- | --- | --- | --- |
|  | Asthma | | | |  | Wheeze | | | |  | Early-onset asthma† | | | |  | Late-onset asthma‡ | | | |
| SNP | OR | 95% CI | P value | FDR |  | OR | 95% CI | P value | FDR |  | OR | 95% CI | P value | FDR |  | OR | 95% CI | P value | FDR |
| rs1800925 |  |  |  |  |  |  |  |  |  |  |  |  |  |  |  |  |  |  |  |
| CC^a^ | 1 |  |  |  |  | 1 |  |  |  |  | 1 |  |  |  |  | 1 |  |  |  |
| CT^a^ | 1.1 | (0.8,1.5) | 0.56 | 0.90 |  | 1.2 | (1.0,1.5) | 0.13 | 0.17 |  | 1.1 | (0.8,1.6) | 0.54 | 0.67 |  | 1.1 | (0.7,1.8) | 0.70 | 0.67 |
| TT^a^ | 1.6 | (0.9,3.0) | 0.16 | 0.62 |  | 2.0 | (1.2,3.3) | 0.01 | 0.04 |  | 1.7 | (0.8,3.6) | 0.20 | 0.67 |  | 1.7 | (0.6,4.8) | 0.35 | 0.41 |
| CT or TT^b^ | 1.2 | (0.9,1.5) | 0.34 | 0.75 |  | 1.3 | (1.0,1.6) | 0.03 | 0.12 |  | 1.2 | (0.8,1.6) | 0.35 | 0.56 |  | 1.2 | (0.7,1.9) | 0.53 | 0.67 |
|  |  |  |  |  |  |  |  |  |  |  |  |  |  |  |  |  |  |  |  |
| rs2066960 |  |  |  |  |  |  |  |  |  |  |  |  |  |  |  |  |  |  |  |
| CC^a^ | 1 |  |  |  |  | 1 |  |  |  |  | 1 |  |  |  |  | 1 |  |  |  |
| CA^a^ | 1.0 | (0.8,1.3) | 0.99 | 0.97 |  | 0.9 | (0.7,1.2) | 0.43 | 0.47 |  | 1.2 | (0.9,1.7) | 0.27 | 0.67 |  | 0.8 | (0.5,1.2) | 0.17 | 0.41 |
| AA^a^ | 0.7 | (0.5,1.0) | 0.06 | 0.44 |  | 0.7 | (0.5,0.9) | 0.02 | 0.04 |  | 0.8 | (0.5,1.3) | 0.30 | 0.67 |  | 0.5 | (0.3,1.0) | 0.05 | 0.40 |
| CA or AA^b^ | 0.9 | (0.7,1.2) | 0.50 | 0.75 |  | 0.9 | (0.7,1.1) | 0.12 | 0.16 |  | 1.1 | (0.8,1.5) | 0.62 | 0.62 |  | 0.7 | (0.5,1.1) | 0.06 | 0.24 |
|  |  |  |  |  |  |  |  |  |  |  |  |  |  |  |  |  |  |  |  |
| rs20541 |  |  |  |  |  |  |  |  |  |  |  |  |  |  |  |  |  |  |  |
| CC^a^ | 1 |  |  |  |  | 1 |  |  |  |  | 1 |  |  |  |  | 1 |  |  |  |
| CT^a^ | 1.0 | (0.8,1.3) | 0.97 | 0.97 |  | 1.1 | (0.9,1.4) | 0.47 | 0.47 |  | 1.1 | (0.8,1.6) | 0.46 | 0.67 |  | 0.8 | (0.5,1.3) | 0.26 | 0.41 |
| TT^a^ | 1.3 | (0.8,1.9) | 0.28 | 0.75 |  | 1.5 | (1.1,2.1) | 0.02 | 0.04 |  | 1.2 | (0.7,2.0) | 0.58 | 0.67 |  | 1.5 | (0.8,2.8) | 0.20 | 0.41 |
| CT or T^b^ | 1.1 | (0.8,1.3) | 0.75 | 0.75 |  | 1.2 | (1.0,1.4) | 0.16 | 0.16 |  | 1.1 | (0.8,1.5) | 0.42 | 0.56 |  | 0.9 | (0.6,1.4) | 0.63 | 0.67 |
|  |  |  |  |  |  |  |  |  |  |  |  |  |  |  |  |  |  |  |  |
| rs848 |  |  |  |  |  |  |  |  |  |  |  |  |  |  |  |  |  |  |  |
| GG^a^ | 1 |  |  |  |  | 1 |  |  |  |  | 1 |  |  |  |  | 1 |  |  |  |
| GT^a^ | 1.0 | (0.8,1.3) | 0.82 | 0.97 |  | 1.2 | (1.0,1.5) | 0.12 | 0.17 |  | 1.2 | (0.8,1.6) | 0.39 | 0.67 |  | 0.8 | (0.5,1.2) | 0.36 | 0.41 |
| TT^a^ | 1.2 | (0.8,1.7) | 0.44 | 0.87 |  | 1.3 | (1.0,1.9) | 0.08 | 0.16 |  | 1.1 | (0.7,1.8) | 0.78 | 0.78 |  | 1.3 | (0.7,2.5) | 0.35 | 0.41 |
| GT or TT^b^ | 1.1 | (0.8,1.4) | 0.65 | 0.75 |  | 1.2 | (1.0,1.5) | 0.06 | 0.12 |  | 1.1 | (0.8,1.5) | 0.41 | 0.56 |  | 0.9 | (0.6,1.4) | 0.67 | 0.67 |
| Models are adjusted for age, sex, parental history of asthma, parental history of atopy, *in utero* exposures to maternal smoking, ETS, dampness, incense burning, pet ownership at home and community. | | | | | | | | | | | | | | | | | | | |
| †Early-onset: asthma diagnosed ≦5 yr of age. | | | | | | | | | | | | | | | | | | | |
| ‡Late-onset: asthma diagnosed >5 yr of age. | | | | | | | | | | | | | | | | | | | |
| ^a^ co-dominant model | | | | | | | | | | | | | | | | | | | |
| ^b^ dominant model | | | | | | | | | | | | | | | | | | | |
