# Supplementary material for: Interleukin-13 Genetic Variants, Household Carpet Use and Childhood Asthma
Source: PLoS One. 2013 Jan 30;8(1):e51970. doi: 10.1371/journal.pone.0051970 (PMC3559736; doi:10.1371/journal.pone.0051970)
Supplement: Table S4 — Environmental questions of TCHS questionnaire. (DOC) [file pone.0051970.s004.doc]

| Table S4. Environmental questions of TCHS questionnaire | | |
| --- | --- | --- |
| 1. Does this child ever exposed to second-hand smoke at home (someone smoked inside this child’s home)? 2. None; (2) Yes; (3) Yes, but didn’t during the past 12 months | | |
| If yes, then:   1. On average, how many cigarettes are smoked inside this child’s home each day? | | |
| 1. Less than 10 cigarettes (1/2 pack) | | 1. 11~20 cigarettes (1/2 pack~1 pack) |
| 1. 21~40 cigarettes (1 pack~2 packs) | | 1. 41~60 cigarettes (2 packs~3packs) |
| 1. More than 60 cigarettes (3 packs) | |  |
|  | |  |
| If yes, then:   1. How much percent of exposing to second-hand smoke during this child’s lifetime? | | |
| 1. Less than 20% | | 1. 21%~40% |
| 1. 41%~60% | | 1. 61%~80% |
| 1. More than 81% | |  |
|  | |  |
| 1. Do you ever have any dog in your home? 2. None; (2) Yes; (3) Yes, but didn’t during the past 12 months | | |
| 1. Do you ever have any cat in your home? 2. None; (2) Yes; (3) Yes, but didn’t during the past 12 months | | |
| 1. Do you ever have any other pets or animals in your home? 2. None; (2) Yes; (3) Yes, but didn’t during the past 12 months | | |
| 1. Have you ever used carpets in your house? 2. None; (2) Yes; (3) Yes, but didn’t during the past 12 months   If yes, then in what rooms? (answer all that apply)   1. Living room 2. Children’s bedroom 3. Other bedroom(s) | | |
| 1. Have you ever perceived visible mould in the walls or bathroom in your house? 2. None; (2) Yes; (3) Yes, but didn’t during the past 12 months | | |
| If yes, then how many walls with visible mould in your house? | | |
| 1. 1 wall | 1. 2 walls | |
| 1. 3 walls | 1. More than four walls | |
|  |  | |
| 1. Have you ever perceived mould odor in your house? 2. None; (2) Yes; (3) Yes, but didn’t during the past 12 months | | |
| If yes, then how often? | | |
| 1. Seldom | 1. 1~3 times per month | |
| 1. 1~3 times per week | 1. Daily | |
|  |  | |
| 1. Have you ever perceived wet stamps because of moist in the ceilings, floors or walls in your house? 2. None; (2) Yes; (3) Yes, but didn’t during the past 12 months | | |
| If yes, then how many walls with water stamps in your house? | | |
| 1. 1 wall | 1. 2 walls | |
| 1. 3 walls | 1. More than four walls | |
|  | | |
| 1. Have you ever had water damage in your house? 2. None; (2) Yes; (3) Yes, but didn’t during the past 12 months | | |
| If yes, then how often? | | |
| 1. 1 day | 1. 1~7 days | |
| 1. 7~30 days | 1. 1~3 months | |
| 1. More than 3 months |  | |
|  | | |
| 1. How often and how much incense is burned in the household when the child stayed in the room? 2. None; (2) Yes; (3) Yes, but didn’t during the past 12 months   If yes, then how often?   1. Less than daily (2 times per month or at major festivals) 2. Daily (all day long everyday and morning and night everyday) | | |
