# Supplementary material for: Interleukin-13 Genetic Variants, Household Carpet Use and Childhood Asthma
Source: PLoS One. 2013 Jan 30;8(1):e51970. doi: 10.1371/journal.pone.0051970 (PMC3559736; doi:10.1371/journal.pone.0051970)
Supplement: Table S5 — Association of household carpet use and ETS at home with asthma phenotypes among children. (DOC) [file pone.0051970.s005.doc]

| Table S5. Association of household carpet use and ETS at home with asthma phenotypes among children | | | | | |
| --- | --- | --- | --- | --- | --- |
|  | Household carpet use | |  | ETS at home | |
|  | OR | 95% CI |  | OR | 95% CI |
| Asthma | 0.8 | (0.5,1.3) |  | 0.9 | (0.7,1.2) |
| Wheeze | 1.0 | (0.7,1.4) |  | 1.1 | (0.9,1.3) |
| Early-onset asthma† | 0.4 | (0.2,1.0) |  | 1.0 | (0.7,1.3) |
| Late-onset asthma‡ | 1.8 | (1.0,3.3) |  | 0.9 | (0.6,1.4) |
| Models are adjusted for age, sex, parental history of asthma, parental history of atopy, *in utero* exposures to maternal smoking, dampness, incense burning, pet ownership at home and community. | | | | | |
| †Early-onset: asthma diagnosed ≦5 yr of age | | | | | |
| ‡Late-onset: asthma diagnosed >5 yr of age. | | | | | |
