# Supplementary material for: Interleukin-13 Genetic Variants, Household Carpet Use and Childhood Asthma
Source: PLoS One. 2013 Jan 30;8(1):e51970. doi: 10.1371/journal.pone.0051970 (PMC3559736; doi:10.1371/journal.pone.0051970)
Supplement: Table S6 — Joint effects of ETS exposure and IL-13 haplotype h1011 on asthma phenotypes among children. (DOC) [file pone.0051970.s006.doc]

| Table S6. Joint effects of ETS exposure and *IL-13* haplotype h1011 on asthma phenotypes among children | | | | | | | | |
| --- | --- | --- | --- | --- | --- | --- | --- | --- |
|  |  |  | h1011^a^ | | | | |  |
|  |  |  | No | |  | Yes | |  |
|  | ETS at home |  | OR | 95%CI |  | OR | 95%CI | P for interaction |
| Asthma | No |  | 1 |  |  | 1.1 | (0.8,1.6) | 0.79 |
|  | Yes |  | 1.0 | (0.7,1.3) |  | 1.1 | (0.7,1.7) |  |
| Wheeze | No |  | 1 |  |  | 1.3 | (0.9,1.8) | 0.82 |
|  | Yes |  | 1.1 | (0.9,1.4) |  | 1.5 | (1.1,2.1) |  |
| Early-onset asthma† | No |  | 1 |  |  | 1.0 | (0.6,1.6) | 0.39 |
|  | Yes |  | 0.9 | (0.7,1.3) |  | 1.3 | (0.8,2.1) |  |
| Late-onset asthma‡ | No |  | 1 |  |  | 1.3 | (0.7,2.4) | 0.65 |
|  | Yes |  | 1.0 | (0.6,1.6) |  | 1.0 | (0.5,2.2) |  |
| Models are adjusted for age, sex, parental history of asthma, parental history of atopy, *in utero* exposures to maternal smoking, dampness, incense burning, pet ownership at home and community. | | | | | | | | |
| †Early-onset: asthma diagnosed ≦5 yr of age. | | | | | | | | |
| ‡Late-onset: asthma diagnosed >5 yr of age. | | | | | | | | |
| ^a^0: common allele and 1: minor allele, by the order of SNP1 (rs1800925): C/T; SNP2 (rs2066960): C/A; SNP3 (rs20541): C/T; SNP4 (rs848): G/T. | | | | | | | | |
